# Pulmonary Emphysema in Cystic Fibrosis Detected by Densitometry on Chest Multidetector Computed Tomography

**DOI:** 10.1371/journal.pone.0073142

**Published:** 2013-08-21

**Authors:** Mark O. Wielpütz, Oliver Weinheimer, Monika Eichinger, Matthias Wiebel, Jürgen Biederer, Hans-Ulrich Kauczor, Claus P. Heußel, Marcus A. Mall, Michael Puderbach

**Affiliations:** 1 Department of Diagnostic and Interventional Radiology, University of Heidelberg, Heidelberg, Germany; 2 Translational Lung Research Center (TLRC) Heidelberg, Member of the German Center for Lung Research (DZL), Heidelberg, Germany; 3 Department of Diagnostic and Interventional Radiology, Medicine of Johannes Gutenberg-University, Mainz, Germany; 4 Department of Radiology, German Cancer Research Center (DKFZ), Heidelberg, Germany; 5 Department of Pulmonology, Cystic Fibrosis Center, Thoraxklinik at University of Heidelberg, Heidelberg, Germany; 6 Department of Diagnostic and Interventional Radiology with Nuclear Medicine, Thoraxklinik at University of Heidelberg, Heidelberg, Germany; 7 Department of Translational Pulmonology and Division of Pediatric Pulmonology and Cystic Fibrosis Center, University of Heidelberg, Heidelberg, Germany; Johns Hopkins School of Medicine, United States of America

## Abstract

**Background:**

Histopathological studies on lung specimens from patients with cystic fibrosis (CF) and recent results from a mouse model indicate that emphysema may contribute to CF lung disease. However, little is known about the relevance of emphysema in patients with CF. In the present study, we used computationally generated density masks based on multidetector computed tomography (MDCT) of the chest for non-invasive characterization and quantification of emphysema in CF.

**Methods:**

Volumetric MDCT scans were acquired in parallel to pulmonary function testing in 41 patients with CF (median age 20.1 years; range 7-66 years) and 21 non-CF controls (median age 30.4 years; range 4-68 years), and subjected to dedicated software. The lung was segmented, low attenuation volumes below a threshold of -950 Hounsfield units were assigned to emphysema volume (EV), and the emphysema index was computed (EI). Results were correlated with forced expiratory volume in 1 s percent predicted (FEV1%), residual volume (RV), and RV/total lung capacity (RV/TLC).

**Results:**

We show that EV was increased in CF (457±530 ml) compared to non-CF controls (78±90 ml) (*P*<0.01). EI was also increased in CF (7.7±7.5%) compared to the control group (1.2±1.4%) (*P*<0.05). EI correlated inversely with FEV1% (r_s_=-0.66), and directly with RV (r_s_=0.69) and RV/TLC (r_s_=0.47) in patients with CF (*P*<0.007), but not in non-CF controls. Emphysema in CF was detected from early adolescence (~13 years) and increased with age (r_s_=0.67, *P*<0.001).

**Conclusions:**

Our results indicate that early onset emphysema detected by densitometry on chest MDCT is a characteristic pathology that contributes to airflow limitation and may serve as a novel endpoint for monitoring lung disease in CF.

## Introduction

Cystic fibrosis (CF) lung disease is caused by mutations in the cystic fibrosis transmembrane conductance regulator (*CFTR*) gene and is the most common genetic form of chronic obstructive pulmonary disease (COPD) [1,2]. CFTR malfunction results in airway surface dehydration and impaired mucociliary clearance leading to airway mucus obstruction, neutrophilic inflammation and bacterial infection [3–5]. It is well established that this pathogenic sequence lead to early onset bronchiectasis that contributes to progressive loss of lung function and disease burden in patients with CF [5–7].

Histopathological studies in necropsy specimens from patients with CF performed in the 1960s to 1980s also reported structural changes in the peripheral airways consistent with emphysema [8–10]. Our previous studies in mice with airway-specific overexpression of the β-subunit of the epithelial Na^+^ channel (ENaC) demonstrated that CF-like airway surface dehydration does not only cause chronic mucus obstruction and inflammation, but also emphysema [11–14]. Further, recent studies showed that cigarette smoke decreases CFTR expression and function [15,16], and that CFTR protein expression correlates inversely with emphysema severity in lungs from patients with cigarette smoke-induced COPD suggesting that impaired CFTR function may be implicated in emphysema formation in humans [17]. However, in contrast to COPD, where emphysema has long been recognized as an important phenotype [18], limited imaging data on emphysema in CF is available [19,20] and the clinical relevance of emphysema in CF remains largely unknown.

Multidetector computed tomography (MDCT) of the chest is widely used for the quantification of emphysema in cigarette smoke-induced COPD and α1-antytrypsin deficiency [21,22], employing density masks generated by dedicated post-processing tools based on Hounsfield units (HU). Previous MDCT imaging and histomorphological studies of lung parenchyma defined a threshold density of -950 HU on inspiratory MDCT and demonstrated that values below this density are diagnostic for emphysema and correlate well with loss of lung function in COPD [21,23,24]. Further, previous studies also demonstrated that MDCT allows to distinguish emphysema from air-trapping [25–27].

Based on previous histopathological studies [8–10], potential pathophysiological commonalities with cigarette-smoke induced COPD [15–17] and our own results from a mouse model of CF lung disease [11–14], we hypothesized that emphysema is present and contributes to airflow limitation in patients with CF. To test this hypothesis, we used MDCT of the chest as a non-invasive method to study the frequency and severity of emphysema in CF. Emphysema indices were determined from thin-section MDCT employing computationally generated density masks and results obtained for CF patients were compared with non-CF controls. To study the relationship between emphysema and lung function, emphysema severity was correlated with pulmonary function testing (PFT). Finally, emphysema severity was correlated with age to determine the onset and progression of emphysema in patients with CF.

## Materials and Methods

### Ethics Statement

The study was carried out as a retrospective analysis of clinically indicated MDCT performed between April 2003 and January 2012 and has been approved by the Ethics Committee of the Medical Faculty of the University of Heidelberg. Informed written consent for examination and further data processing was obtained from patients or legal guardians.

### Study Population

Table 1 provides a summary of the clinical characteristics of our study population. The diagnosis of CF was established by clinical symptoms characteristic of CF, increased sweat Cl^-^ concentrations and/or detection of disease causing mutations in the *CFTR* gene as previously described [28]. The *CFTR* genotypes of CF patients are provided in the online supplement (Table S1). All CF patients showed characteristic signs of CF lung disease such as bronchial wall thickening, mucus plugging and bronchiectasis of at least one lobe. The non-CF control group was recruited from non-smoking patients who obtained a diagnostic chest MDCT for various indications but showed no evidence of airway disease, emphysema or major parenchymal changes upon reading of the diagnostic MDCT scan. Additional information is provided in the online supplement (Methods S1).

**Table 1:** Characteristics of study population.

	**CONTROL**	**CF**
Age [a]	30.4 (4-68)	20.1 (7-66)
Sex	13 ♂ / 8 ♀	22 ♂ / 19 ♀
MDCT n =	21	41
PFT n =	15	39
ΔPFT-MDCT [d]	1 (0 - 66)	0 (0 - 73)
FEV1 [l]	3.7 ± 0.9	1.6 ± 1.2^†^
FEV1%	102 ± 16	46 ± 30^†^
VC [l]	4.3 ± 1.0	2.4 ± 1.3^†^
VC%	100 ± 15	64 ± 23^†^
RV [l]	1.8 ± 0.6	2.8 ± 1.5*
RV%	107 ± 26	192 ± 71^†^
TLC [l]	5.9 ± 0.8	5.3 ± 2.1
TLC%	101 ± 9	103 ± 12

Summary of age, gender and lung function data from patients with cystic fibrosis (CF) and non-CF controls (CONTROL), who underwent multidetector computed tomography (MDCT) and pulmonary function testing (PFT), including forced expiratory volume in 1 s (FEV1), vital capacity (VC), residual volume (RV), and total lung capacity (TLC). Data given as mean or median ± SD or with data range in brackets as appropriate. * *P*<0.05, † *P*<0.001.

### Multidetector Computed Tomography

Non-enhanced MDCT at end-inspiratory breath-hold in supine position and thin-section reconstructions with a medium soft kernel algorithm were performed as previously described [21,29]. Further details are provided in the online supplement (Methods S1).

### Quantitative MDCT Densitometry

The MDCT images were analyzed using a custom in-house software (YACTA) as previously described, and controlled for extra-corporal air attenuation [29,30]. After the segmentation of the lung from the stack of MDCT images, a lung voxel was assigned to emphysema if its density was equal to or below the threshold of -950 HU, as routinely used for the quantification of emphysema in COPD [21,31]. The volume of the segmented lung (LV) and emphysema (EV), EV/LV ratio (pixel index = emphysema index, EI), lung weight (LW), mean lung density in HU (MLD) and the 15^th^ percentile of the density histogram (15th) were calculated automatically. 15th is defined as the threshold value in HU for which 15% of lung voxels have a lower density. A manual correction of the results was carried out to exclude sacculations, abscesses, cysts or bronchiectases from emphysema voxels (Figure S1). This step became necessary in most CF patients and took around 15 min per patient. Additional information on densitometry is provided in the online supplement (Methods S1).

### Pulmonary Function Testing

The following lung function parameters (absolute and percent predicted values) acquired by whole-body plethysmography were chosen for correlation analysis: forced expiratory volume in 1 s (FEV1, FEV1%), vital capacity (VC, VC%), FEV1 to VC ratio (FEV1/VC, “Tiffeneau index”), residual volume (RV, RV%), total lung capacity (TLC, TLC%). To estimate the degree of hyperinflation, the RV to TLC ratio was calculated (RV/TLC). Additional information is provided in the online supplement (Methods S1 and Figure S2).

### Statistical Analysis

Data were analyzed using SigmaPlot® (Systat Software GmbH, Erkrath, Germany). Groups were compared by Student’s t-test or Wilcoxon rank sum test, and the Pearson r (absolute values) or Spearman rank order correlation coefficient r_s_ (EI, percent predicted values) were calculated for selected MDCT vs. PFT parameters as appropriate. A *P*-value of <0.05 or <0.05/m (number of tests) with Bonferroni’s method to correct for multiple testing was accepted to indicate statistical significance [32].

## Results

### Detection of emphysema in patients with CF by MDCT

**Figure 1:**
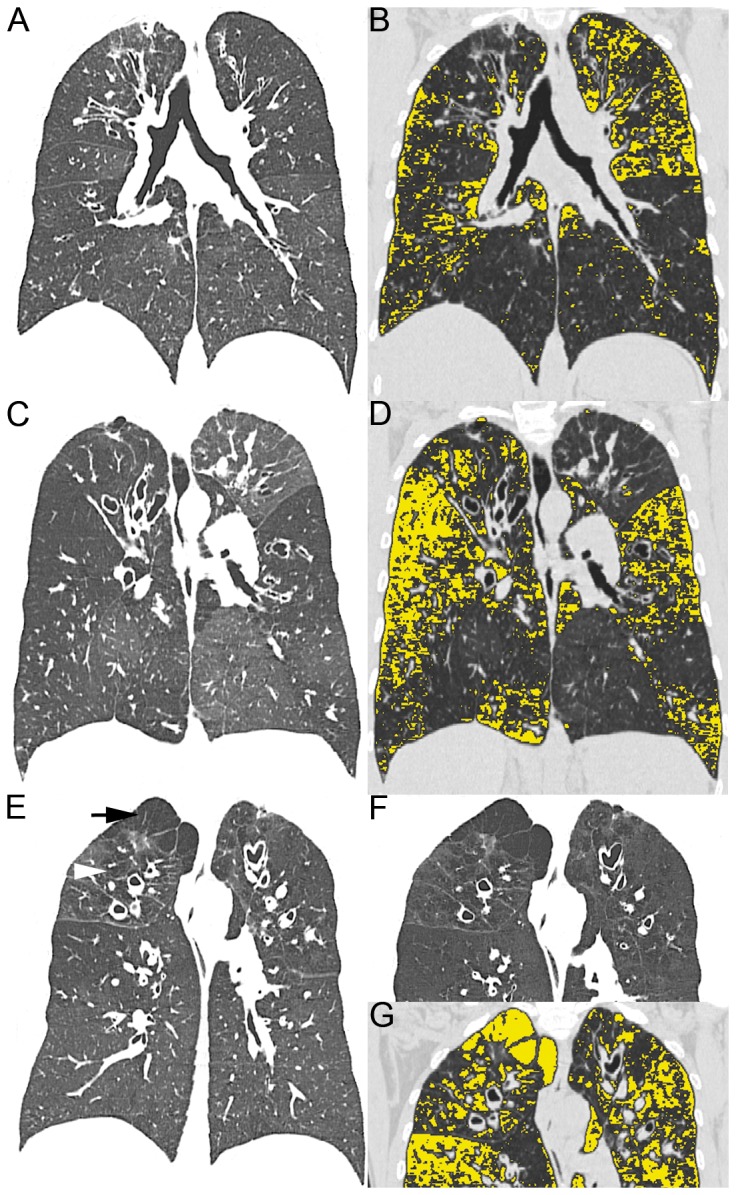
Visualization of emphysema distribution in cystic fibrosis (CF) patients by chest MDCT density masks. (A–G) Representative examples of morphologic images from non-enhanced multidetector computed tomography (MDCT) of the chest (left panels A, C, E) are complemented by density maps generated by dedicated software highlighting low attenuation areas below -950 Hounsfield units (HU) in yellow (right panels B, D, G). (A,B) MDCT image of a 36 year-old female CF patient with FEV1% = 48% showing bronchiectasis (A) as well as hypodense areas corresponding to emphysema (EI = 13.2%) mainly along subpleural and bronchovascular structures (B). (C,D) 38 year-old male CF patient with FEV1% = 29% with the lung parenchyma of the upper segments of the inferior lobes showing an overall hypodense texture and constricted vasculature (C). The density map shows extensive emphysema (EI = 24.0%) of both lungs with an emphasis on the lower lobes (D). (E–G) 46 year-old male cystic fibrosis patient (FEV1% = 55%) with marked bullous paraseptal emphysema of the right lung apex (black arrow) and centrilobular emphysema predominantly of both upper lobes (white arrowhead) (E). Note that these bullae do not possess walls differentiating them from cysts or sacculations (compare Figure S1). The minimum intensity projection (MinIP, 5 mm slice thickness) emphasizes emphysema visualization by accentuating low attenuation areas (F) with an overall EI of 18.0% (G).

Different patterns of emphysematous lesions were observed in CF patients with increasing age and severity of lung disease (Figure 1). In young CF patients with a low EI, emphysema voxels were mainly observed in the subpleural regions. With increasing EI, more voxels were found along bronchovascular structures with an emphasis on the lung periphery (Figure 1A,B). High EI resulted in extensive involvement of the parenchyma with a spread to the perihilar region (Figure 1C,D). Some CF patients with advanced lung disease showed a centrilobular and paraseptal emphysema pattern (Figure 1E–G).

### Quantification of emphysema in CF lung disease

**Figure 2:**
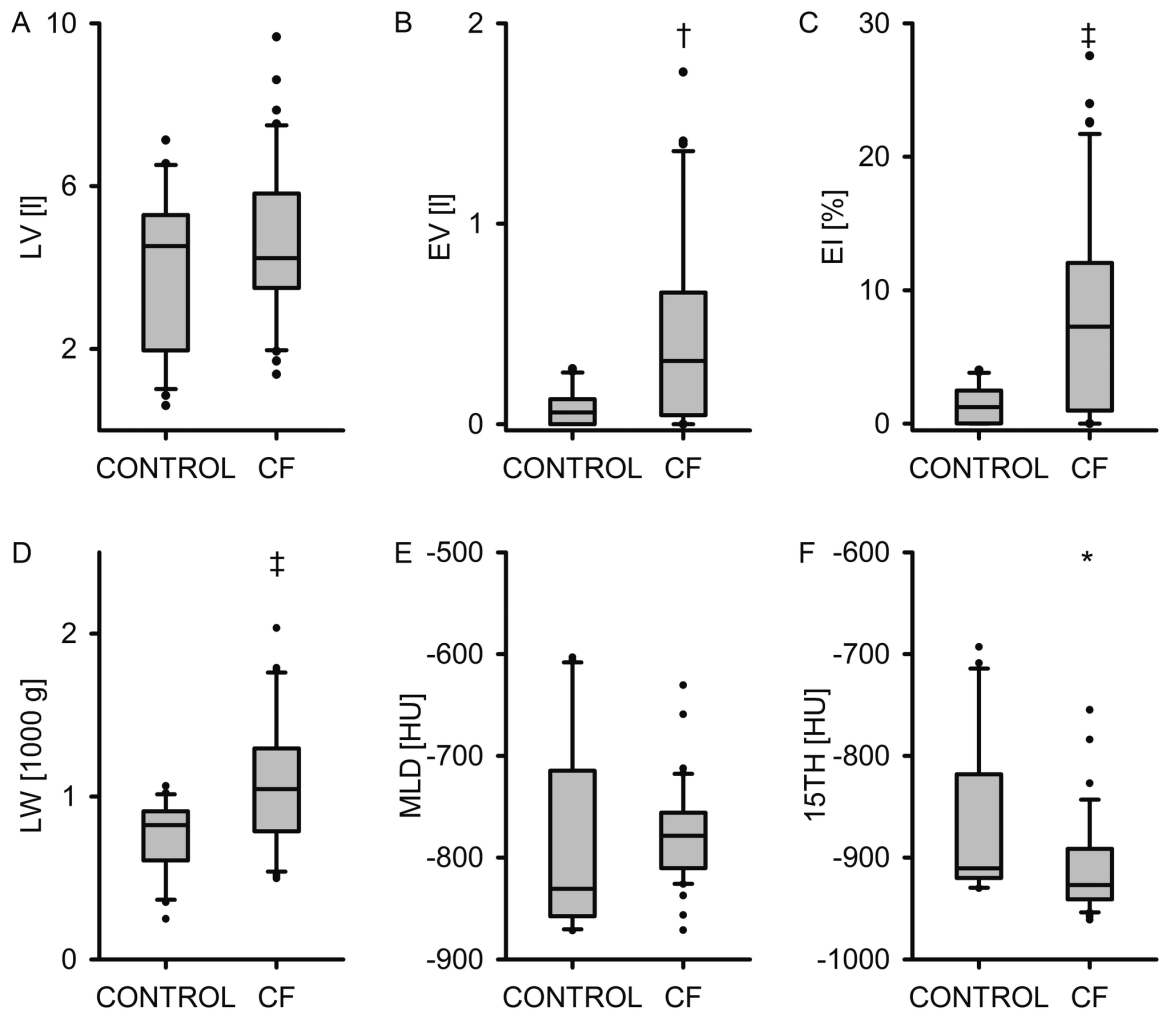
Quantification of emphysema in cystic fibrosis (CF) lung disease by densitometry. (A–F) Box-and-whisker plots for lung volume (LV) (A), emphysema volume (EV) (B) and emphysema index (EI) (C), lung weight (LW) (D), mean lung density (MLD) (E) and 15^th^ percentile of the lung density histogram (15th) (F) in the non-CF control group (CONTROL) and patients with CF. The central line represents the median, the box encompasses the 25^th^-75^th^ percentiles, whiskers show 10^th^ and 90^th^ percentiles, and closed circles (•) represent individual outliers. * *P*<0.05, † *P*<0.01 and ‡ *P*<0.001 compared to CONTROL.

For quantification of emphysema in our CF study population, we next determined LV, EV, EI, LW, MLD and 15th, and compared values obtained from CF patients with non-CF controls (Figure 2). These quantitative analyses of the density masks demonstrated that LV remained unchanged (Figure 2A), but that EV (*P*<0.01) and EI (*P*<0.001) were significantly increased in CF patients compared to non-CF controls (Figure 2B,C). LW was also increased in CF (*P*<0.001), probably due to areas of increased density, e.g. due to mucus or inflammation, whereas MLD was not different in CF compared to non-CF controls (Figure 2D,E). Finally, 15th was significantly reduced in CF patients versus controls (*P*<0.05) (Figure 2F). Taken together, these results identify emphysema as a characteristic lesion in CF lung disease.

### Correlation between emphysema severity and lung function in CF

**Figure 3:**
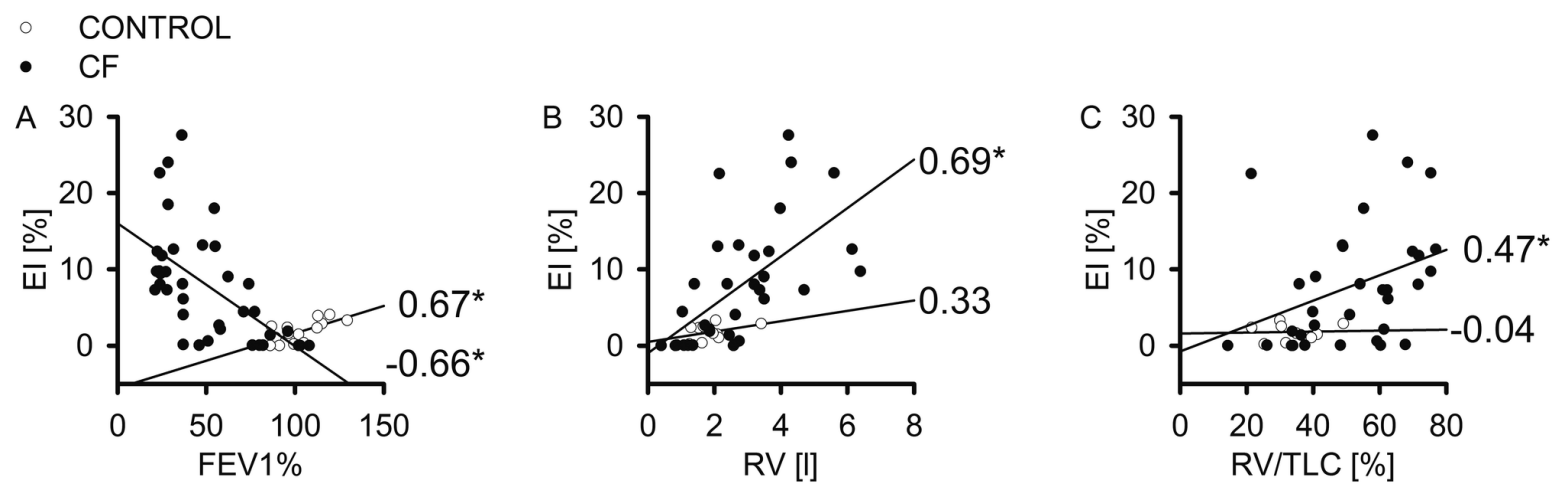
Emphysema severity correlates with impairment in lung function in cystic fibrosis (CF). (A–C) Dot plots with linear regression curves for emphysema index (EI) plotted against forced expiratory volume in 1 s percent predicted (FEV1%) (A), residual volume (RV) (B), and RV as ratio of total lung capacity (RV/TLC) (C) for patients with CF and the non-CF control group (CONTROL). Spearman rank order correlation coefficients (r_s_) are given for each plot. * *P*<0.05/7 (Bonferroni’s method, see Table 2).

Next, we studied the correlation between quantitative emphysema indices, as determined from MDCT densitometry and pulmonary function (Tab. 2 and Figure 3). As shown in Figure 3, EI showed a significant inverse correlation with FEV1% (r_s_ = -0.66, *P*<0.05/7) (Figure 3A), i.e. r_s_^2^ = 43% of the decrease in FEV1% may be explained by variations in EI. Further, EI was directly correlated with total RV as well as RV/TLC (Figure 3B,C). This relationship between lung density and lung function was also confirmed by significant correlations of 15th with FEV1%, RV and RV/TLC in CF, but not in non-CF controls (Tab. 2). These results indicate that emphysema contributes to airflow limitation in CF.

**Table 2:** Correlation analysis of densitometry with lung function.

		**FEV1**	**FEV1%**	**VC**	**FEV1/VC**	**RV**	**TLC**	**RV/TLC**
**CONTROL**	LV	0.67	0.75*	0.67	-0.14	0.16	0.79*	-0.38
	LW	0.47	0.61*	0.40	0.19	0.13	0.29	-0.02
	EV	0.67	0.71*	0.70	-0.04	0.37	0.83*	-0.13
	EI	0.55	0.67*	0.62	0.01	0.33	0.78*	-0.04
	MLD	-0.47	-0.62*	-0.54	0.10	-0.37	-0.66	0.15
	15th	-0.48	-0.70*	-0.52	0.00	-0.39	-0.67	0.14
**CF**	LV	0.29	-0.46*	0.50*	-0.56*	0.75*	0.92*	0.37
	LW	0.15	-0.44*	0.35	-0.49*	0.80*	0.88*	0.35
	EV	0.24	-0.55*	0.40	-0.65*	0.59*	0.73*	0.48*
	EI	-0.27	-0.66*	0.05	-0.63*	0.69*	0.61*	0.47*
	MLD	-0.09	0.22	-0.23	0.38	-0.42	-0.46*	-0.19
	15th	0.16	0.56*	-0.01	0.64*	-0.62*	-0.49*	-0.50*

Summary of correlation analyses between densitometry on chest multidetector computed tomography (MDCT) and pulmonary function testing in patients with cystic fibrosis (CF) and non-CF control subjects (CONTROL). Pearson r or Spearman r_s_ rank order coefficient were calculated for lung volume (LV), lung weight (LW), emphysema volume (EV), emphysema index (EI), mean lung density (MLD), and 15^th^ percentile of lung density (15th) with forced expiratory volume within 1 s (FEV1, FEV1%), vital capacity (VC), Tiffeneau index (FEV1/VC), residual volume (RV), total lung capacity (TLC), and RV/TLC ratio. * *P*<0.05/7 (Bonferroni’s method, 7 tests per MDCT parameter).

### Timing of onset and progression of emphysema in CF lung disease

**Figure 4:**
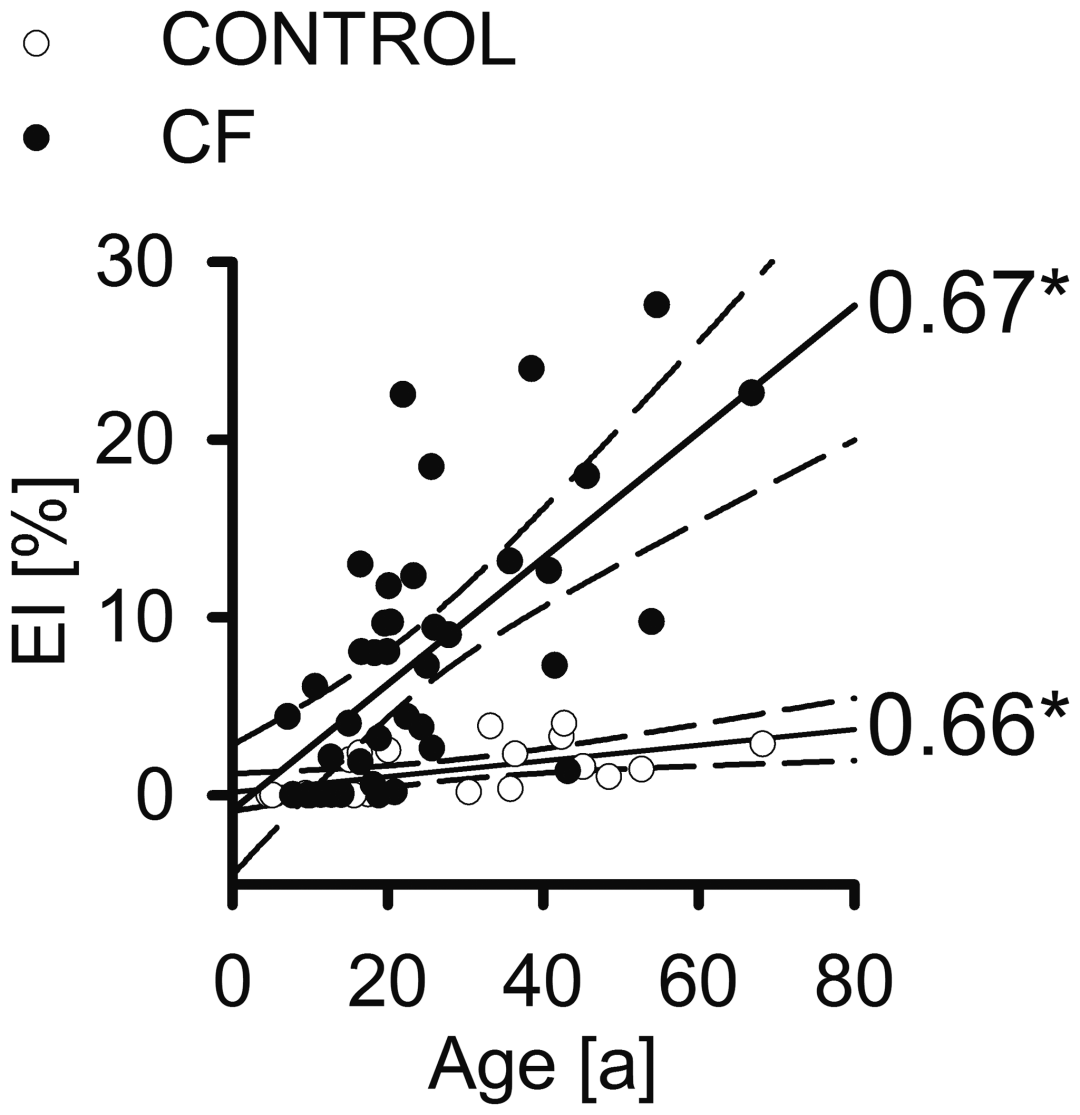
Emphysema progresses with age in cystic fibrosis (CF). Dot plots with linear regression curves for emphysema index (EI) plotted against patient age for patients with CF and the non-CF control group (CONTROL). Spearman rank order correlation coefficients (r_s_) are given for each plot. Dashed curves indicate 95% confidence intervals. * *P*<0.001.

Plotting the EI against age demonstrated that normal lung ageing was associated with a small increase of EI in individuals from the non-CF control group. In the CF group, emphysema severity correlated significantly with patient age, and the slope of incline with age was significantly larger in CF (regression slope of 0.35) compared to non-CF controls (regression slope 0.04) (*P*<0.0001) (Figure 4). The limits of the 95% confidence intervals for regression curves obtained from CF patients and non-CF controls intersected at ~13 years of age (Figure 4). These results suggest that the majority of CF patients develop significant emphysema beyond this threshold age.

## Discussion

Emphysema is a major disease phenotype that determines the morbidity and mortality of many patients with cigarette smoke-induced COPD [33]. Although it is well established that CF and COPD share key features including small airways mucus obstruction and chronic pulmonary inflammation [5,34], little is known about the frequency of occurrence and clinical relevance of emphysema in patients with CF [8,35,36]. Besides histopathological post-mortem studies [8–10], emphysema was depicted in CF in some previous MDCT imaging studies including a semi-quantitative visual scoring system developed for assessment of morphological changes of the CF lung [19,20]. However, these studies did not report any quantitative or densitometric data on emphysema. Furthermore, subsequent work did not further assess the relevance of emphysema, but rather focused on the development of bronchiectasis [37,38] and the contribution of air-trapping to ventilation impairment [39,40] in CF lung disease. Air-trapping results in regional hypoperfusion on inspiratory MDCT, and may be diagnosed more sensitively by paired inspiratory/expiratory MDCT [40]. Of note, previous imaging studies in patients with COPD demonstrated that air-trapping is associated with a density range between -860 and -950 HU, which is higher than the density threshold defining emphysema [41].

In phenotyping of patients with COPD, MDCT has long been accepted for the visual and computational quantification of emphysema [21,31,42], including recent large epidemiological trials (COPDGene, ECLIPSE) [43,44]. In the present study, we demonstrate that CF patients develop significant emphysema in addition to airway mucus plugging and bronchiectasis (Fig. 1). Using MDCT densitometry as a non-invasive method with a threshold value of -950 HU, and indices well established for the diagnosis and quantification of emphysema in patients with cigarette smoke-induced COPD [31], emphysema in CF patients was evidenced by a significant increase in EV and EI (Figures 1 and 2). Similar to previous studies in patients with COPD [26,29], emphysema severity in CF correlated significantly with airflow limitation and hyperinflation, as determined from FEV1%, RV and RV/TLC (Figure 3). Based on this correlation, we estimate that on average, emphysema accounted for ~43% of FEV1% reduction in the CF patients included in our study. These results show that emphysema is a clinically relevant phenotype contributing to the severity of lung disease in a subgroup of patients with CF. Further, our results suggest that chest MDCT densitometry might be a suitable non-invasive method for the diagnosis and quantitative monitoring of emphysema progression in individual patients with CF.

Compared to patients with COPD, overall emphysema severity was moderate in our cross-sectional study in children and mostly young adults with CF [29,45,46]. Values for EV and EI were on average less elevated in CF compared to the values previously reported for patients with advanced stages of COPD. Further, the mean MLD, often used as an emphysema marker in COPD, did not differ and the estimated lung weight (LW) was increased rather than reduced in patients with CF compared to non-CF controls (Figure 2). We speculate that normal MLD and elevated LW in CF may result from areas with increased density due to regional mucus retention, inflammation and/or compensatory hyperperfusion, which may all hamper the use of MLD and LW as emphysema parameters in CF. However, the values for 15th of lung density were significantly reduced in CF compared to age-matched non-CF controls (Figure 2). Taken together, these results support the notion that lesions with elevated tissue density and emphysema coexist in the CF lung, and suggest that the EI and 15th may be more reliable than MLD in estimating emphysema severity in CF.

Correlating the EI with age demonstrated that, in contrast to common early lesions of the conducting airways such as mucus obstruction associated with air-trapping, airway wall thickening and bronchiectasis [7,47], emphysema is rarely present in children with CF (Figure 4). However, emphysema formation was observed in early adolescence (~13 years of age) and emphysema severity progressed in adult patients with CF (Figure 4). In contrast, consistent with previous reports in healthy adults, little emphysema was observed in non-CF controls (Figures 2 and 4) [48]. This timing of occurrence and progression shows that early onset emphysema is a characteristic feature of CF lung disease, and suggests that emphysema develops secondary to chronic airways disease in patients with CF. The clinical relevance of this phenotype is highlighted by an increase in life expectancy of patients with CF with a median survival of ~40 years in North America and Western Europe [49,50].

In COPD, emphysema pathogenesis with structural damage and remodeling of distal airspaces has been linked to cigarette smoke-induced oxidative stress, inflammation, extracellular matrix proteolysis, alveolar cell death, and disrupted alveolar maintenance triggering apoptosis and autophagy [51]. We speculate that CFTR dysfunction may trigger several of these mechanisms and thereby induce emphysema formation in patients with CF. First, it is well established that airway surface dehydration caused by CFTR malfunction in airway epithelia is an important disease mechanisms that impairs mucociliary clearance and triggers the pathogenetic cascade of airway mucus obstruction, chronic inflammation and bacterial infection in CF lung disease [3–5]. Our previous studies in βENaC-overexpressing mice demonstrated that mucus obstruction and airway inflammation caused by airway surface dehydration are associated with emphysema formation *in vivo* with increased lung volumes, distal airspace enlargement, increased lung compliance and reduced density of lung parenchyma, as determined from volumetric CT studies [12,14,52]. Recent studies indicate that airway surface dehydration causes impaired *in vivo* clearance of inhaled particulates and bacterial products such as lipopolysaccharide (LPS), which trigger the recruitment of macrophages and neutrophils, and increase secretion of elastolytic proteases such as macrophage elastase (matrix metalloprotease 12) and neutrophil elastase into the airspaces [53–55]. Hence, similar to cigarette smoke-induced COPD [56], proteolytic damage of distal airspaces due to a protease/antiprotease imbalance caused by proteases released in chronic inflammation, may also play an important role in emphysema formation in CF. A second link between CFTR dysfunction and emphysema formation was suggested by recent studies demonstrating i) that cigarette smoke exposure reduces CFTR expression and function [15,16,57] *in vitro* and *in vivo*; ii) that CFTR protein levels correlate inversely with ceramide accumulation and emphysema severity in lungs from COPD patients [17]; and iii) that CFTR controls cigarette-smoke induced apoptosis and autophagy in mice [58]. These studies suggest that, in addition to airway surface dehydration and mucostasis caused by impaired CFTR Cl^-^ channel function, CFTR dysfunction may cause other abnormalities on the cellular level, such as altered ceramide metabolism, that may play an important role in alveolar inflammation and emphysema formation in CF [59–61]. However, further studies are required to determine the relative role of these mechanisms for emphysema formation in patients with CF.

In addition to further mechanistic studies on emphysema pathophysiology, it will also be important to assess the relationship between *CFTR* genotypes, as well as treatment regimens, and emphysema development in CF [1,5,28]. Due to the limited number of patients available for analysis, we were not able to address these issues in this retrospective study. Hence, future longitudinal studies in larger patient cohorts are necessary to determine the impact of different classes of CFTR mutations, differences in treatment regimens and adherence to therapy, as well as other environmental and genetic factors on emphysema in patients with CF.

In summary, we demonstrate that early onset and progressive emphysema is a characteristic feature of CF lung disease. Emphysema severity determined by chest MDCT correlated with airflow limitation, suggesting MDCT densitometry as a non-invasive method for detection and monitoring of emphysema progression in individual patients with CF. Our results also suggest that emphysema contributes to disease severity and may therefore serve as a novel endpoint for monitoring of lung disease in patients with CF.

## Supporting Information

Figure S1 **Necessity of manual adaptation of density maps.** Coronary reconstructions of a multidetector computed tomogram of the chest of a 22 year-old female cystic fibrosis patient without density map (A), with the density map (emphysema depicted in yellow color) generated by the automatic software algorithm (B), and after manual adaptation to exclude cystic lesions and bronchiectasis in the right superior lobe (black arrows). Emphysema severity may be overestimated by the automatic software algorithm, if they are not connected to the airway tree or airway segmentation was interrupted. The emphysema index of the right lung was calculated as 15.3% without manual correction (B) and 13.6% after manual correction (C).
(TIF)

Figure S2 **Validation of segmented lung volume from inspiratory computed tomography (CT) against pulmonary function testing.** Dot plot with linear regression curve for lung volume (LV) determined from CT images plotted against total lung capacity (TLC) as derived from whole-body plethysmography. Data from cystic fibrosis (CF) patients are shown as closed circles and data from non-CF controls (CONTROL) as open circles. The Pearson correlation coefficient (r) for pooled analysis is indicated. * *P*<0.001.
(TIF)

Table S1 ***CFTR* genotypes of patients with CF.**
(DOC)

Methods S1 **Supplementary methods section.**
(DOC)

## References

[1] Welsh MJ, Ramsey B, Accurso F, Cutting GR. Cystic fibrosis (2001) Cystic fibrosis; Scriver CR, Beaudet AL, Sly WS, Valle D, editorsThe Metabolic & Molecular Bases of Inherited Disease. 8th ed. New York: McGraw-Hill pp. 5121-5188.

[2] Riordan JR, Rommens JM, Kerem B, Alon N, Rozmahel R et al. (1989) Identification of the cystic fibrosis gene: cloning and characterization of complementary DNA. Science 245: 1066-1073. doi:10.1126/science.2475911. PubMed: 2475911. 2475911 10.1126/science.2475911

[3] Boucher RC (2007) Airway surface dehydration in cystic fibrosis: pathogenesis and therapy. Annu Rev Med 58: 157-170. doi:10.1146/annurev.med.58.071905.105316. PubMed: 17217330. 17217330 10.1146/annurev.med.58.071905.105316

[4] Mall MA (2008) Role of cilia, mucus, and airway surface liquid in mucociliary dysfunction: lessons from mouse models. J Aerosol Med Pulm Drugs Deliv 21: 13-24. doi:10.1089/jamp.2007.0659. PubMed: 18518828. 10.1089/jamp.2007.0659 18518828

[5] Gibson RL, Burns JL, Ramsey BW (2003) Pathophysiology and management of pulmonary infections in cystic fibrosis. Am J Respir Crit Care Med 168: 918-951. doi:10.1164/rccm.200304-505SO. PubMed: 14555458. 14555458 10.1164/rccm.200304-505SO

[6] Stick SM, Brennan S, Murray C, Douglas T, von Ungern-Sternberg BS et al. (2009) Bronchiectasis in infants and preschool children diagnosed with cystic fibrosis after newborn screening. J Pediatr 155: 623-628. doi:10.1016/j.jpeds.2009.05.005. PubMed: 19616787. 19616787 10.1016/j.jpeds.2009.05.005

[7] de Jong PA, Nakano Y, Hop WC, Long FR, Coxson HO et al. (2005) Changes in airway dimensions on computed tomography scans of children with cystic fibrosis. Am J Respir Crit Care Med 172: 218-224. doi:10.1164/rccm.200410-1311OC. PubMed: 15831838. 15831838 10.1164/rccm.200410-1311OC

[8] Esterly JR, Oppenheimer EH (1968) Cystic fibrosis of the pancreas: structural changes in peripheral airways. Thorax 23: 670-675. doi:10.1136/thx.23.6.670. PubMed: 5711776. 5711776 10.1136/thx.23.6.670 PMC471884

[9] Bedrossian CW, Greenberg SD, Singer DB, Hansen JJ, Rosenberg HS (1976) The lung in cystic fibrosis. A quantitative study including prevalence of pathologic findings among different age groups. Hum Pathol 7: 195-204. doi:10.1016/S0046-8177(76)80023-8. PubMed: 1262016. 1262016 10.1016/s0046-8177(76)80023-8

[10] Sobonya RE, Taussig LM (1986) Quantitative aspects of lung pathology in cystic fibrosis. Am Rev Respir Dis 134: 290-295. PubMed: 3740655. 3740655 10.1164/arrd.1986.134.2.290

[11] Mall M, Grubb BR, Harkema JR, O’Neal WK, Boucher RC (2004) Increased airway epithelial Na^+^ absorption produces cystic fibrosis-like lung disease in mice. Nat Med 10: 487-493. doi:10.1038/nm1028. PubMed: 15077107. 15077107 10.1038/nm1028

[12] Mall MA, Harkema JR, Trojanek JB, Treis D, Livraghi A et al. (2008) Development of chronic bronchitis and emphysema in β-epithelial Na^+^ channel-overexpressing mice. Am J Respir Crit Care Med 177: 730-742. doi:10.1164/rccm.200708-1233OC. PubMed: 18079494. 18079494 10.1164/rccm.200708-1233OC PMC2277210

[13] Mall MA (2009) Role of the amiloride-sensitive epithelial Na^+^ channel in the pathogenesis and as a therapeutic target for cystic fibrosis lung disease. Exp Physiol 94: 171-174. doi:10.1113/expphysiol.2008.042994. PubMed: 19060118. 19060118 10.1113/expphysiol.2008.042994

[14] Wielpütz MO, Eichinger M, Zhou Z, Leotta K, Hirtz S et al. (2011) In vivo monitoring of cystic fibrosis-like lung disease in mice by volumetric computed tomography. Eur Respir J 38: 1060-1070. doi:10.1183/09031936.00149810. PubMed: 21478215. 21478215 10.1183/09031936.00149810

[15] Cantin AM, Hanrahan JW, Bilodeau G, Ellis L, Dupuis A et al. (2006) Cystic fibrosis transmembrane conductance regulator function is suppressed in cigarette smokers. Am J Respir Crit Care Med 173: 1139-1144. doi:10.1164/rccm.200508-1330OC. PubMed: 16497995. 16497995 10.1164/rccm.200508-1330OC

[16] Clunes LA, Davies CM, Coakley RD, Aleksandrov AA, Henderson AG et al. (2012) Cigarette smoke exposure induces CFTR internalization and insolubility, leading to airway surface liquid dehydration. FASEB J 26: 533-545. doi:10.1096/fj.11-192377. PubMed: 21990373. 21990373 10.1096/fj.11-192377 PMC3290447

[17] Bodas M, Min T, Mazur S, Vij N (2011) Critical modifier role of membrane-cystic fibrosis transmembrane conductance regulator-dependent ceramide signaling in lung injury and emphysema. J Immunol 186: 602-613. doi:10.4049/jimmunol.1002850. PubMed: 21135173. 21135173 10.4049/jimmunol.1002850 PMC3119853

[18] Han MK, Agusti A, Calverley PM, Celli BR, Criner G et al. (2010) Chronic obstructive pulmonary disease phenotypes: the future of COPD. Am J Respir Crit Care Med 182: 598-604. doi:10.1164/rccm.200912-1843CC. PubMed: 20522794. 20522794 10.1164/rccm.200912-1843CC PMC6850732

[19] Helbich TH, Heinz-Peer G, Eichler I, Wunderbaldinger P, Götz M et al. (1999) Cystic fibrosis: CT assessment of lung involvement in children and adults. Radiology 213: 537-544. PubMed: 10551238. 10551238 10.1148/radiology.213.2.r99nv04537

[20] Helbich TH, Heinz-Peer G, Fleischmann D, Wojnarowski C, Wunderbaldinger P et al. (1999) Evolution of CT findings in patients with cystic fibrosis. AJR Am J Roentgenol 173: 81-88. doi:10.2214/ajr.173.1.10397104. PubMed: 10397104. 10397104 10.2214/ajr.173.1.10397104

[21] Coxson HO, Rogers RM (2005) Quantitative computed tomography of chronic obstructive pulmonary disease. Acad Radiol 12: 1457-1463. doi:10.1016/j.acra.2005.08.013. PubMed: 16253858. 16253858 10.1016/j.acra.2005.08.013

[22] Ley-Zaporozhan J, van Beek EJ (2010) Imaging phenotypes of chronic obstructive pulmonary disease. J Magn Reson Imaging 32: 1340-1352. doi:10.1002/jmri.22376. PubMed: 21105139. 21105139 10.1002/jmri.22376

[23] Gevenois PA, De Vuyst P, de Maertelaer V, Zanen J, Jacobovitz D et al. (1996) Comparison of computed density and microscopic morphometry in pulmonary emphysema. Am J Respir Crit Care Med 154: 187-192. doi:10.1164/ajrccm.154.1.8680679. PubMed: 8680679. 8680679 10.1164/ajrccm.154.1.8680679

[24] Coxson HO, Rogers RM, Whittall KP, D’Yachkova Y, Paré PD et al. (1999) A quantification of the lung surface area in emphysema using computed tomography. Am J Respir Crit Care Med 159: 851-856. doi:10.1164/ajrccm.159.3.9805067. PubMed: 10051262. 10051262 10.1164/ajrccm.159.3.9805067

[25] Barbosa EM Jr., Song G, Tustison N, Kreider M, Gee JC et al. (2011) Computational analysis of thoracic multidetector row HRCT for segmentation and quantification of small airway air trapping and emphysema in obstructive pulmonary disease. Acad Radiol 18: 1258-1269. doi:10.1016/j.acra.2011.06.004. PubMed: 21893294. 21893294 10.1016/j.acra.2011.06.004

[26] Mets OM, Murphy K, Zanen P, Gietema HA, Lammers JW et al. (2012) The relationship between lung function impairment and quantitative computed tomography in chronic obstructive pulmonary disease. Eur Radiol 22: 120-128. doi:10.1007/s00330-011-2237-9. PubMed: 21837396. 21837396 10.1007/s00330-011-2237-9 PMC3229695

[27] Galbán CJ, Han MK, Boes JL, Chughtai KA, Meyer CR et al. (2012) Computed tomography-based biomarker provides unique signature for diagnosis of COPD phenotypes and disease progression. Nat Med 18: 1711-1715. doi:10.1038/nm.2971. PubMed: 23042237. 23042237 10.1038/nm.2971 PMC3493851

[28] Hirtz S, Gonska T, Seydewitz HH, Thomas J, Greiner P et al. (2004) CFTR Cl^-^ channel function in native human colon correlates with the genotype and phenotype in cystic fibrosis. Gastroenterology 127: 1085-1095. doi:10.1053/j.gastro.2004.07.006. PubMed: 15480987. 15480987 10.1053/j.gastro.2004.07.006

[29] Heussel CP, Herth FJ, Kappes J, Hantusch R, Hartlieb S et al. (2009) Fully automatic quantitative assessment of emphysema in computed tomography: comparison with pulmonary function testing and normal values. Eur Radiol 19: 2391-2402. doi:10.1007/s00330-009-1437-z. PubMed: 19458953. 19458953 10.1007/s00330-009-1437-z

[30] Wielpütz MO, Eichinger M, Weinheimer O, Ley S, Mall MA et al. (2013) Automatic Airway Analysis on Multidetector Computed Tomography in Cystic Fibrosis: Correlation With Pulmonary Function Testing. J Thorac Imaging 28: 104-113. doi:10.1097/RTI.0b013e3182765785. PubMed: 23222199. 23222199 10.1097/RTI.0b013e3182765785

[31] Coxson HO, Mayo J, Lam S, Santyr G, Parraga G et al. (2009) New and current clinical imaging techniques to study chronic obstructive pulmonary disease. Am J Respir Crit Care Med 180: 588-597. doi:10.1164/rccm.200901-0159PP. PubMed: 19608719. 19608719 10.1164/rccm.200901-0159PP

[32] Curtin F, Schulz P (1998) Multiple correlations and Bonferroni’s correction. Biol Psychiatry 44: 775-777. doi:10.1016/S0006-3223(98)00043-2. PubMed: 9798082. 9798082 10.1016/s0006-3223(98)00043-2

[33] Rabe KF, Hurd S, Anzueto A, Barnes PJ, Buist SA et al. (2007) Global strategy for the diagnosis, management, and prevention of chronic obstructive pulmonary disease: GOLD executive summary. Am J Respir Crit Care Med 176: 532-555. doi:10.1164/rccm.200703-456SO. PubMed: 17507545. 17507545 10.1164/rccm.200703-456SO

[34] Hogg JC, Chu F, Utokaparch S, Woods R, Elliott WM et al. (2004) The nature of small-airway obstruction in chronic obstructive pulmonary disease. N Engl J Med 350: 2645-2653. doi:10.1056/NEJMoa032158. PubMed: 15215480. 15215480 10.1056/NEJMoa032158

[35] Bhalla M, Turcios N, Aponte V, Jenkins M, Leitman BS et al. (1991) Cystic fibrosis: scoring system with thin-section CT. Radiology 179: 783-788. PubMed: 2027992. 2027992 10.1148/radiology.179.3.2027992

[36] Robinson TE (2004) High-resolution CT scanning: potential outcome measure. Curr Opin Pulm Med 10: 537-541. doi:10.1097/01.mcp.0000142924.38801.45. PubMed: 15510063. 15510063 10.1097/01.mcp.0000142924.38801.45

[37] Brody AS, Kosorok MR, Li Z, Broderick LS, Foster JL et al. (2006) Reproducibility of a scoring system for computed tomography scanning in cystic fibrosis. J Thorac Imaging 21: 14-21. doi:10.1097/01.rti.0000203937.82276.ce. PubMed: 16538150. 16538150 10.1097/01.rti.0000203937.82276.ce

[38] de Jong PA, Lindblad A, Rubin L, Hop WC, de Jongste JC et al. (2006) Progression of lung disease on computed tomography and pulmonary function tests in children and adults with cystic fibrosis. Thorax 61: 80-85. PubMed: 16244089. 16244089 10.1136/thx.2005.045146 PMC2080716

[39] Brody AS, Tiddens HA, Castile RG, Coxson HO, de Jong PA et al. (2005) Computed tomography in the evaluation of cystic fibrosis lung disease. Am J Respir Crit Care Med 172: 1246-1252. doi:10.1164/rccm.200503-401PP. PubMed: 16100011. 16100011 10.1164/rccm.200503-401PP

[40] Hall GL, Logie KM, Parsons F, Schulzke SM, Nolan G et al. (2011) Air trapping on chest CT is associated with worse ventilation distribution in infants with cystic fibrosis diagnosed following newborn screening. PLOS ONE 6: e23932. doi:10.1371/journal.pone.0023932. PubMed: 21886842. 21886842 10.1371/journal.pone.0023932 PMC3158781

[41] Matsuoka S, Kurihara Y, Yagihashi K, Hoshino M, Watanabe N et al. (2008) Quantitative assessment of air trapping in chronic obstructive pulmonary disease using inspiratory and expiratory volumetric MDCT. AJR Am J Roentgenol 190: 762-769. doi:10.2214/AJR.07.2820. PubMed: 18287450. 18287450 10.2214/AJR.07.2820

[42] Hoffman EA, Simon BA, McLennan G (2006) State of the Art. A structural and functional assessment of the lung via multidetector-row computed tomography: phenotyping chronic obstructive pulmonary disease. Proc Am Thorac Soc 3: 519-532. doi:10.1513/pats.200603-086MS. PubMed: 16921136. 16921136 10.1513/pats.200603-086MS PMC2647643

[43] Vestbo J, Anderson W, Coxson HO, Crim C, Dawber F et al. (2008) Evaluation of COPD Longitudinally to Identify Predictive Surrogate End-points (ECLIPSE). Eur Respir J 31: 869-873. doi:10.1183/09031936.00111707. PubMed: 18216052. 18216052 10.1183/09031936.00111707

[44] Regan EA, Hokanson JE, Murphy JR, Make B, Lynch DA et al. (2010) Genetic epidemiology of COPD (COPDGene) study design. COPD 7: 32-43. doi:10.3109/15412550903499522. PubMed: 20214461. 20214461 10.3109/15412550903499522 PMC2924193

[45] Han MK, Kazerooni EA, Lynch DA, Liu LX, Murray S et al. (2011) Chronic obstructive pulmonary disease exacerbations in the COPDGene study: associated radiologic phenotypes. Radiology 261: 274-282. doi:10.1148/radiol.11110173. PubMed: 21788524. 21788524 10.1148/radiol.11110173 PMC3184233

[46] Martinez CH, Chen YH, Westgate PM, Liu LX, Murray S et al. (2012) Relationship between quantitative CT metrics and health status and BODE in chronic obstructive pulmonary disease. Thorax 67: 399-406. doi:10.1136/thoraxjnl-2011-201185. PubMed: 22514236. 22514236 10.1136/thoraxjnl-2011-201185 PMC3719874

[47] Sly PD, Brennan S, Gangell C, de Klerk N, Murray C et al. (2009) Lung disease at diagnosis in infants with cystic fibrosis detected by newborn screening. Am J Respir Crit Care Med 180: 146-152. doi:10.1164/rccm.200901-0069OC. PubMed: 19372250. 19372250 10.1164/rccm.200901-0069OC

[48] Zach JA, Newell JD Jr., Schroeder J, Murphy JR, Curran-Everett D et al. (2012) Quantitative computed tomography of the lungs and airways in healthy nonsmoking adults. Invest Radiol 47: 596-602. doi:10.1097/RLI.0b013e318262292e. PubMed: 22836310. 22836310 10.1097/RLI.0b013e318262292e PMC3703944

[49] Dodge JA, Lewis PA, Stanton M, Wilsher J (2007) Cystic fibrosis mortality and survival in the UK: 1947-2003. Eur Respir J 29: 522-526. doi:10.1183/09031936.00099506. PubMed: 17182652. 17182652 10.1183/09031936.00099506

[50] Stern M, Wiedemann B, Wenzlaff P (2008) From registry to quality management: the German Cystic Fibrosis Quality Assessment project 1995-2006. Eur Respir J 31: 29-35. doi:10.1183/09031936.00056507. PubMed: 17898017. 17898017 10.1183/09031936.00056507

[51] Tuder RM, Petrache I (2012) Pathogenesis of chronic obstructive pulmonary disease. J Clin Invest 122: 2749-2755. doi:10.1172/JCI60324. PubMed: 22850885. 22850885 10.1172/JCI60324 PMC3408733

[52] Zhou Z, Duerr J, Johannesson B, Schubert SC, Treis D et al. (2012) The ENaC-overexpressing mouse as a model of cystic fibrosis lung disease. J Cyst Fibros 10 Suppl 2: S172-S182. PubMed: 21658636. 10.1016/S1569-1993(11)60021-0 21658636

[53] Cobos-Correa A, Trojanek JB, Diemer S, Mall MA, Schultz C (2009) Membrane-bound FRET probe visualizes MMP12 activity in pulmonary inflammation. Nat Chem Biol 5: 628-630. doi:10.1038/nchembio.196. PubMed: 19648933. 19648933 10.1038/nchembio.196

[54] Schubert S, Trojanek J, Diemer S, Cobos A, Zhou Z et al. (2009) Airways surface liquid depletion causes MMP-12 dependent emphysema in βENaC-overexpressing mice [abstract] J Cyst Fibros 8: S53. doi:10.1016/S1569-1993(09)60212-5.

[55] Gaggar A, Hector A, Bratcher PE, Mall MA, Griese M et al. (2011) The role of matrix metalloproteinases in cystic fibrosis lung disease. Eur Respir J 38: 721-727. doi:10.1183/09031936.00173210. PubMed: 21233269. 21233269 10.1183/09031936.00173210 PMC4036453

[56] Hautamaki RD, Kobayashi DK, Senior RM, Shapiro SD (1997) Requirement for macrophage elastase for cigarette smoke-induced emphysema in mice. Science 277: 2002-2004. doi:10.1126/science.277.5334.2002. PubMed: 9302297. 9302297 10.1126/science.277.5334.2002

[57] Alexander NS, Blount A, Zhang S, Skinner D, Hicks SB et al. (2012) Cystic fibrosis transmembrane conductance regulator modulation by the tobacco smoke toxin acrolein. Laryngoscope 122: 1193-1197. doi:10.1002/lary.23278. PubMed: 22522920. 22522920 10.1002/lary.23278 PMC3548450

[58] Bodas M, Min T, Vij N (2011) Critical role of CFTR-dependent lipid rafts in cigarette smoke-induced lung epithelial injury. Am J Physiol Lung Cell Mol Physiol 300: L811-L820. doi:10.1152/ajplung.00408.2010. PubMed: 21378025. 21378025 10.1152/ajplung.00408.2010 PMC3119127

[59] Petrache I, Natarajan V, Zhen L, Medler TR, Richter AT et al. (2005) Ceramide upregulation causes pulmonary cell apoptosis and emphysema-like disease in mice. Nat Med 11: 491-498. doi:10.1038/nm1238. PubMed: 15852018. 15852018 10.1038/nm1238 PMC1352344

[60] Teichgräber V, Ulrich M, Endlich N, Riethmüller J, Wilker B et al. (2008) Ceramide accumulation mediates inflammation, cell death and infection susceptibility in cystic fibrosis. Nat Med 14: 382-391. doi:10.1038/nm1748. PubMed: 18376404. 18376404 10.1038/nm1748

[61] Ulrich M, Worlitzsch D, Viglio S, Siegmann N, Iadarola P et al. (2010) Alveolar inflammation in cystic fibrosis. J Cyst Fibros 9: 217-227. doi:10.1016/j.jcf.2010.03.001. PubMed: 20347403. 20347403 10.1016/j.jcf.2010.03.001 PMC2883667

