# Supplementary figures and images for: Pulmonary Emphysema in Cystic Fibrosis Detected by Densitometry on Chest Multidetector Computed Tomography
Source: PLoS One. 2013 Aug 21;8(8):e73142. doi: 10.1371/journal.pone.0073142 (PMC3749290; doi:10.1371/journal.pone.0073142)

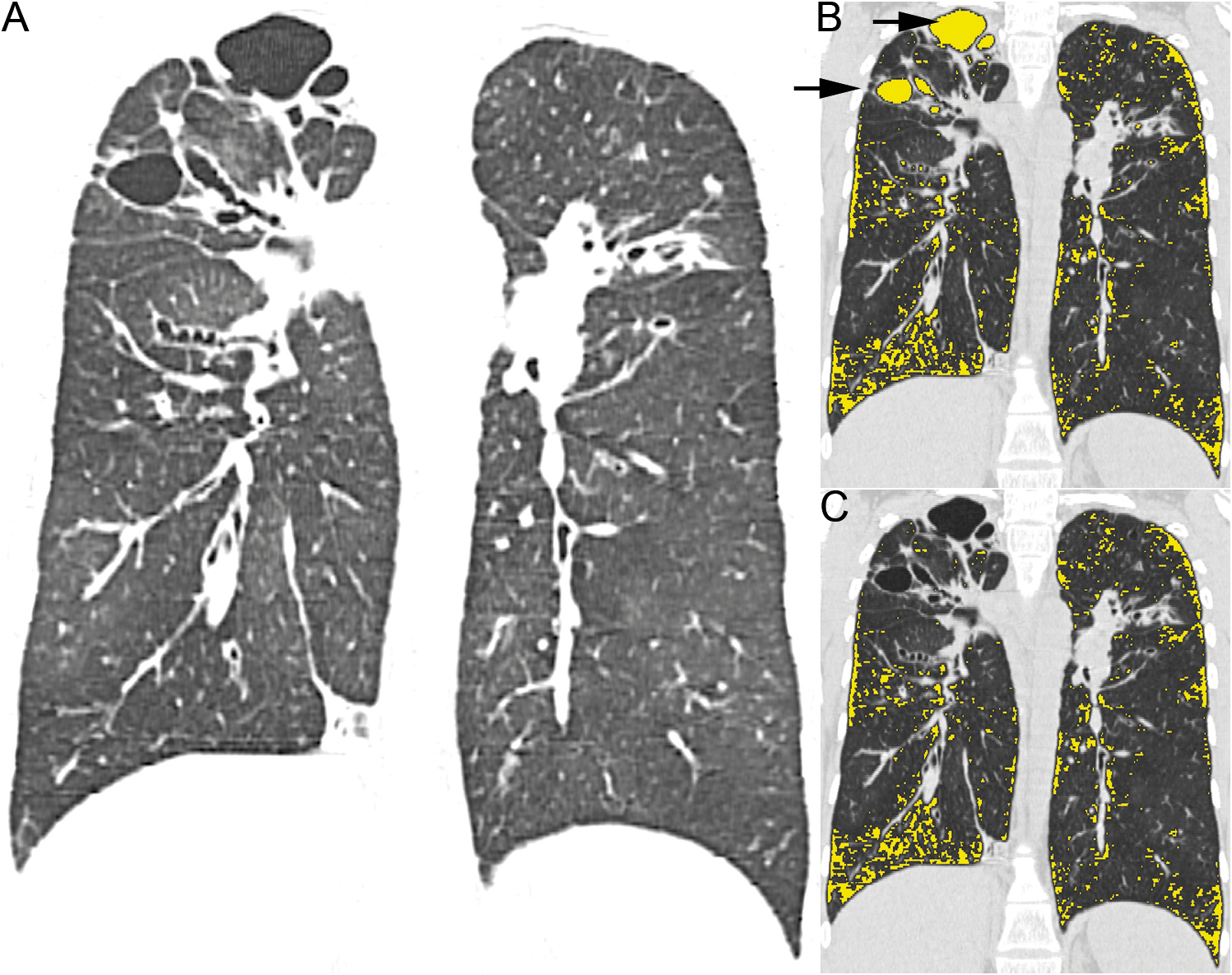

Supplement: Figure S1 — Necessity of manual adaptation of density maps. Coronary reconstructions of a multidetector computed tomogram of the chest of a 22 year-old female cystic fibrosis patient without density map (A), with the density map (emphysema depicted in yellow color) generated by the automatic software algorithm (B), and after manual adaptation to exclude cystic lesions and bronchiectasis in the right superior lobe (black arrows). Emphysema severity may be overestimated by the automatic software algorithm, if they are not connected to the airway tree or airway segmentation was interrupted. The emphysema index of the right lung was calculated as 15.3% without manual correction (B) and 13.6% after manual correction (C). (TIF) [file pone.0073142.s001.tif]

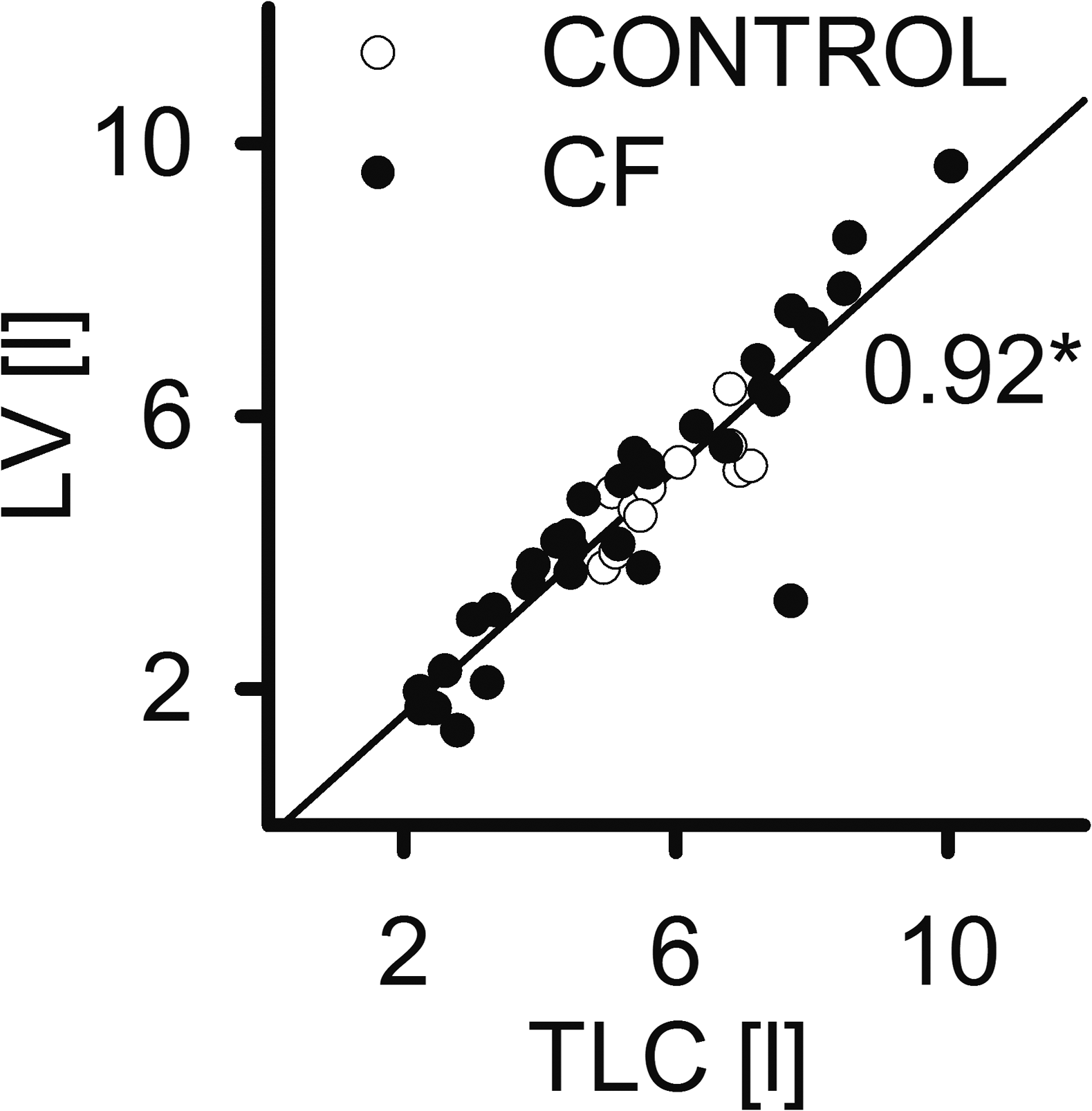

Supplement: Figure S2 — Validation of segmented lung volume from inspiratory computed tomography (CT) against pulmonary function testing. Dot plot with linear regression curve for lung volume (LV) determined from CT images plotted against total lung capacity (TLC) as derived from whole-body plethysmography. Data from cystic fibrosis (CF) patients are shown as closed circles and data from non-CF controls (CONTROL) as open circles. The Pearson correlation coefficient (r) for pooled analysis is indicated. * P<0.001. (TIF) [file pone.0073142.s002.tif]
