# Supplementary material for: Pulmonary Emphysema in Cystic Fibrosis Detected by Densitometry on Chest Multidetector Computed Tomography
Source: PLoS One. 2013 Aug 21;8(8):e73142. doi: 10.1371/journal.pone.0073142 (PMC3749290; doi:10.1371/journal.pone.0073142)
Supplement: Table S1 — CFTR genotypes of patients with CF. (DOC) [file pone.0073142.s003.doc]

| ***CFTR* Genotype** | **Number of Individuals** |
| --- | --- |
|  |  |
| F508del / F508del | 23 |
| F508del / 2183AA->G | 2 |
| F508del / R347P | 2 |
| F508del / W1282X | 2 |
| F508del / I148T | 1 |
| F508del / G542X | 1 |
| F508del / 546insCTA | 1 |
| F508del / 1811+17T>G | 1 |
| F508del / G1244E | 1 |
| F508del / X | 1 |
| R553X / 1717-1G->A | 1 |
| X / X | 5 |
