# Supplementary material for: Pulmonary Emphysema in Cystic Fibrosis Detected by Densitometry on Chest Multidetector Computed Tomography
Source: PLoS One. 2013 Aug 21;8(8):e73142. doi: 10.1371/journal.pone.0073142 (PMC3749290; doi:10.1371/journal.pone.0073142)
Supplement: Methods S1 — Supplementary methods section. (DOC) [file pone.0073142.s004.doc]

**METHODS S1**

**Study Population**

Indications for chest MDCT in the non-CF control group were suspected malignancy (primary or secondary) (n = 7), suspected infection (n = 7), chronic cough and dyspnea (n = 6) and suspected interstitial lung disease (n = 1). Solitary pulmonary nodules < 3 cm in diameter were considered acceptable. Indications for diagnostic chest MDCT in the CF group were assessment of disease severity (n = 39), suspected acute infection (n = 1) and pre-transplantation work-up (n = 1). A spectrum of different ages, and thus a broad variety of stages of CF lung disease were subjected to the software analysis. In two CF patients, a destroyed lobe was evident, which led to the exclusion of the affected lobe from image analysis.

**Multidetector Computed Tomography**

Exclusively non-enhanced thin-section MDCT was routinely performed in supine position [1]. Before scanning, all patients received an instructed training to achieve full end-inspiratory breath-hold (i.e. total lung capacity, TLC). All patients ≥ 18 years were examined with a 4-slice Volume Zoom helical computer tomograph (Siemens AG, Forchheim, Germany) at 120 kV, 70 mAs (effective), a collimation of 1.25 mm, and pitch 2. Some patients < 18 years were examined with a 16-slice Aquillion 16 system (Toshiba Corp., Tokyo, Japan) with a dose-modulated protocol at 120 kV and mAs (effective) adapted to body-weight (range 35 – 80), 1.0 mm collimation, and pitch 1. In total 14 non-CF controls and 31 CF patients were examined with the Siemens, and 7 non-CF controls and 10 patients with CF with the Toshiba system.

Reconstruction was performed with a slice thickness of 1.25 mm and 1.0 mm increment in a medium soft B40f algorithm (Siemens scanner) or with a slice thickness of 1.0 mm and 1.0 mm increment in an equivalent medium soft kernel FC05 (Toshiba scanner), as medium soft kernels were recommended for computational emphysema analysis [2]. The scale of attenuation coefficients with these systems ranges from -1,024 to +3,072 Hounsfield Units (HU). The systems were calibrated for water regularly and after major maintenance, and for air daily. Respiratory artifacts not affecting image analysis were ignored. Examination protocols and equipment were kept constant during the whole inclusion period.

**Quantitative MDCT Densitometry**

Using a custom in-house software (YACTA), the stack of around 300 images per patient was analyzed fully automatically on a standard personal computer (Intel Core 2 Duo 2.26 GHz, 4 GB RAM) in around 5 min per data set, as employed in previous studies [1,3-5]. The initial step is a threshold-based region growing segmentation of the whole lung parenchyma as published previously [6]. The lung volume (LV) is calculated by multiplying lung voxels with voxel volume. Before the creation of density masks, a Gauss filter is applied to each image. Soft tissue (density greater than −750 HU), lung (less than −500 HU), and tracheobronchial tree were identified fully automatically based on threshold values and an anatomical knowledge-based algorithm. A lung voxel was assigned to emphysema if its density equaled or was below the threshold of -950 HU [2,7], with a noise correction for voxels with **−**910 to **−**949HU that needed at least 4 adjacent voxels with a density of **-**950 HU or less. Lung weight (LW) was estimated by a modified algorithm as previously described [8]. A manual correction of the results was carried out to exclude sacculations, abscesses, cysts or bronchiectases from emphysema voxels (Fig. S1). This step became necessary in most CF patients and took around 15 min per patient.

To standardize measurements and to rule out HU drift of air over time, we measured extra-corporal air attenuation in every individual imaging study along the whole z-axis. Median air attenuation for CF was -999 ± 6 HU and for non-CF -999 ± 8 HU, without any differences with regard to the time-point of the examination or patient age. There was a small difference between the Siemens (-999 ±1 HU) and the Toshiba scanner (-989 ± 10 HU). A correction of the emphysema threshold on exams obtained with the Toshiba system by the difference of the individually measured air attenuation from -1000 HU did not lead to different results of LV, EV, EI, LW, MLD or 15TH.

To validate that MDCT analysis is based on full-inspiratory breath-hold, LV as determined from MDCT was correlated with TLC from PFT as the reference standard. These analyses displayed an excellent correlation between LV and TLC in both CF and non-CF individuals, thus validating measurement of LV by MDCT (Fig. S2). Similar to previous results in patients with COPD, TLC was systematically higher than LV in CF (*P*<0.001), suggesting that plethysmography may overestimate TLC in obstructive lung disease [9]. Examination protocols and equipment were kept constant during the whole inclusion period. Computational results were reviewed by a reader with more than 4 years of expertise in chest radiology, preceding their statistical evaluation.

**Pulmonary Function Testing**

Whole-body plethysmography (MasterScreen Body, E. Jaeger, Hoechberg, Germany) was performed according to the guidelines of the European Respiratory Society and the standards of the American Thoracic Society (ATS) [10], and the European Coal and Steal Community (ECSC) predicted values served as the standard of reference [11].

**REFERENCES**

1. Heussel CP, Kappes J, Hantusch R, Hartlieb S, Weinheimer O, et al. (2009) Contrast enhanced CT-scans are not comparable to non-enhanced scans in emphysema quantification. Eur J Radiol 74: 473-478.

2. Coxson HO, Rogers RM (2005) Quantitative computed tomography of chronic obstructive pulmonary disease. Acad Radiol 12: 1457-1463.

3. Heussel CP, Herth FJ, Kappes J, Hantusch R, Hartlieb S, et al. (2009) Fully automatic quantitative assessment of emphysema in computed tomography: comparison with pulmonary function testing and normal values. Eur Radiol 19: 2391-2402.

4. McGregor A, Roberts HC, Dong Z, Menezes R, Kauczor HU, et al. (2010) Repeated low-dose computed tomography in current and former smokers for quantification of emphysema. J Comput Assist Tomogr 34: 933-938.

5. Wielputz MO, Eichinger M, Weinheimer O, Ley S, Mall MA, et al. (2013) Automatic Airway Analysis on Multidetector Computed Tomography in Cystic Fibrosis: Correlation With Pulmonary Function Testing. J Thorac Imaging 28: 104-113.

6. Weinheimer O, Achenbach T, Heussel CP, Düber C (2011) Automatic Lung Segmentation in MDCT Images. In: Proceedings of the Fourth International Workshop on Pulmonary Image Analysis. Toronto. pp. 241-255.

7. Coxson HO, Mayo J, Lam S, Santyr G, Parraga G, et al. (2009) New and current clinical imaging techniques to study chronic obstructive pulmonary disease. Am J Respir Crit Care Med 180: 588-597.

8. Schneider W, Bortfeld T, Schlegel W (2000) Correlation between CT numbers and tissue parameters needed for Monte Carlo simulations of clinical dose distributions. Phys Med Biol 45: 459-478.

9. Garfield JL, Marchetti N, Gaughan JP, Steiner RM, Criner GJ (2012) Total lung capacity by plethysmography and high-resolution computed tomography in COPD. Int J Chron Obstruct Pulmon Dis 7: 119-126.

10. Miller MR, Hankinson J, Brusasco V, Burgos F, Casaburi R, et al. (2005) Standardisation of spirometry. Eur Respir J 26: 319-338.

11. Quanjer PH, Tammeling GJ, Cotes JE, Pedersen OF, Peslin R, et al. (1993) Lung volumes and forced ventilatory flows. Report Working Party Standardization of Lung Function Tests, European Community for Steel and Coal. Official Statement of the European Respiratory Society. Eur Respir J Suppl 16: 5-40.
